# Bread Fortified with Cooked Purple Potato Flour and *Citrus* Albedo: An Evaluation of Its Compositional and Sensorial Properties

**DOI:** 10.3390/foods10050942

**Published:** 2021-04-25

**Authors:** Isabella Taglieri, Chiara Sanmartin, Francesca Venturi, Monica Macaluso, Alessandro Bianchi, Cristina Sgherri, Mike Frank Quartacci, Marinella De Leo, Luisa Pistelli, Fabrizio Palla, Guido Flamini, Angela Zinnai

**Affiliations:** 1 Department of Agriculture Food Environment, University of Pisa, Via Del Borghetto 80, 56124 Pisa, Italy; isabella.taglieri@for.unipi.it (I.T.); chiara.sanmartin@unipi.it (C.S.); monica.macaluso@phd.unipi.it (M.M.); alessandro.bianchi@phd.unipi.it (A.B.); cristina.sgherri@unipi.it (C.S.); mike.frank.quartacci@unipi.it (M.F.Q.); angela.zinnai@unipi.it (A.Z.); 2 Interdepartmental Research Center, Nutraceuticals and Food for Health, University of Pisa, Via del Borghetto 80, 56124 Pisa, Italy; marinella.deleo@unipi.it (M.D.L.); Luisa.pistelli@unipi.it (L.P.); guido.flamini@unipi.it (G.F.); 3 CISUP, Centre for Instrumentation Sharing, University of Pisa, Lungarno Pacinotti 43, 56126 Pisa, Italy; 4 Department of Pharmacy, University of Pisa, Via Bonanno Pisano 6, 56126 Pisa, Italy; 5 INFN, National Institute for Nuclear Physics, Largo Bruno Pontecorvo, 3, 56127 Pisa, Italy; Fabrizio.Palla@cern.ch

**Keywords:** bread, anthocyanins, bioactive compounds, sourdough, baker’s yeast, shelf life, fortification, *Citrus* albedo

## Abstract

This research aimed to explore the feasibility of fortifying bread with cooked Vitelotte potato powder and *Citrus* albedo, comparing the use of baker’s yeast or sourdough as leavening agents. Breads obtained were thus subjected to physico-chemical and sensory characterizations. The replacement of part of the wheat flour with purple potato and albedo determined a significant enhancement of the phenolic profile and antioxidant status of fortified breads, as well as a longer shelf life. Thanks to its acidity and antimicrobial activity, sourdough improved the levels of health-promoting compounds and stability. Both the fortification and the leavening agent deeply affected the organoleptic, expression, and the aroma profile, of the fortified bread. Interestingly, albedo addition, despite its effectiveness in boosting the phenolic profile, determined a higher perception of aftertaste and bitterness, irrespective of the leavening agent. Based on these results, the use of purple potatoes and *Citrus* albedo, if properly formulated, could represent a valuable strategy for the development of high-quality products, with longer shelf-life.

## 1. Introduction

Any food product is doomed to fail after a certain amount of storage time for a variety of reasons: attack by higher organisms, microbial contamination, enzymatic, chemical, and physical degradation. In this context, bread is a dynamic system undergoing physical, chemical and microbiological modifications. Considering that bread is consumed worldwide as a staple food, its staling process limits bread’s shelf life [1,2] and is one of the largest contributions to food waste in the world [3]. In this regard, the use of sourdough as leavening agent provides many technological advantages and higher overall quality over baker’s yeast [4,5,6], including a delayed staling process and the protection of bread from mould and bacterial spoilage [7,8,9,10,11,12,13].

Regarding the rate of staling, also the starch characteristics of the flour used could have a considerable impact on moisture loss and increased firmness [3]. Wheat flour is traditionally used for bread making due to its gluten network structure that confers cohesiveness, extensibility, and elasticity to the dough, also favouring the retention of gas during fermentation [14]. Nowadays, various composite flours are investigated to produce bread with an increased nutraceutical value together with improved physico-chemical properties and extended shelf life [15]. In this context, the addition of potato flour was studied for the potential improvement of the rheological properties of bread. Potato starch, indeed, exhibits a higher swelling power, lower retrogradation as well as higher moisture retention than wheat flour; therefore, bread softness is maintained longer, and bread spoilage is also reduced [16]. Moreover, after cooking and cooling, resistant starch production is favoured, thus increasing the dietary fibre content and slowing postprandial glucose release [17].

Researchers have focused their attention on the possibility to couple the anti-staling effect of potato starch with the antioxidant and antimicrobial activities of phytochemicals contained in some particular potato varieties. Several studies are available on the enrichment of wheat bread with red and purple potato flour, exploiting their high content of bioactive compounds, such as phenolic acids, flavonoids, and among them, anthocyanins, which contains properties and beneficial effects on human health that are widely reported [18,19].

Among purple coloured-fleshed potatoes, many studies have been performed on the Vitelotte potato due to its polyphenolic profile and anthocyanin content [20,21]. Vitelotte potato (*Solanum tuberosum* L.) has been recently taken into consideration as a fortification ingredient to obtain a high nutritional value bread [22]. This ancient potato cultivar is characterized by a dark blue skin and dark violet-blue flesh due to a high content of anthocyanins [22,23]. In breadmaking, the addition of this fortifying agent confers high levels of chlorogenic acid and conjugated myricetin to the product, with a higher antioxidant activity if compared to yellow potato [22]. Moreover, the stability at baking temperature of free and conjugated phenolic acids in a purple-fleshed potato was previously demonstrated [19,24], showing values two and three-fold higher than in a yellow potato [22].

Nowadays, an increasingly popular strategy to avoid microbiological spoilage is represented by the addition of phytochemicals of different food origin, which gives the product a higher oxidative stability and consequently extended shelf life [2,25,26]. The recovery of these bioactive compounds from by-products of the food industry has also become a recent trend for the development of sustainable high value bakery products [27,28]. In this regard, by-products of *Citrus* fruit processing industries are promising sources of materials that may be used in the food industry because of their valuable technological and nutritional properties. Albedo of *Citrus* peel represents an important source of pharmacologically active secondary metabolites, such as flavonoids, which have strong antioxidant, anti-inflammatory, and antimicrobial properties [29,30]. Moreover, albedo is rich in fibres of which the insoluble components, mainly pectins, may constitute up to 25% [31]. Although not commonly applied in breadmaking, pectins were studied for their effect on dough rheology [32] due to the formation of hydrophilic complexes between anionic groups (carboxyl) of pectins and gluten proteins, as well as bread staling delay because of their high water-retention capacity [33,34].

For the above, this research aimed to explore the feasibility of fortifying bread with cooked Vitelotte potato powder and *Citrus* albedo and comparing the use of baker’s yeast or sourdough as leavening agents. Breads obtained were subjected to a physico-chemical and sensory characterizations. A preliminary assessment of the effect of fortification, as well as the choice of the leavening agent, for bread’s shelf life was then carried out.

## 2. Materials and Methods

### 2.1. Chemicals

All chemicals used for the analyses, including standards, methanol, acetonitrile and formic acid, DPPH(2,2-diphenyl-1-picrylhydrazyl) and ABTS (2,2’-azino-bis(3-ethylbenzothiazoline-6-sulfonic acid) were supplied by Sigma Aldrich (Milano, Italy) and Folin–Ciocalteu reagent by Merck (Darmstadt, Germany). A Millipore (Bedford, MA, USA) water purification system was used for Milli-Q water.

### 2.2. Raw Material

The sourdough was maintained by a daily refreshment procedure for one year at the Food Technology laboratory of the Department of Agriculture, Food and Environment (DAFE) of the University of Pisa, while the baker’s yeast was a compressed yeast commercially available (Zeus Iba s.r.l., Firenze, Italy).

The commercial wheat flours utilised for the refreshment (hard flour type 0) and breadmaking (weak flour type 0) were stored at 4 °C until chemical characterization and processing.

Potatoes (*Solanum tuberosum* cv. Vitelotte) were supplied by the Azienda Agricola Ravotto Mauro (Cuneo, Italy) and cultivated at the agro-experimental station of DAFE. After washing, purple potatoes were boiled with the skin to reduce the loss of bioactive compounds as much as possible, and then peeled, sliced and freeze-dried (Lyoquest-55, Telstar, Milano, Italy). The dried chips were then ground into flour using a commercial blender, with intervals to prevent over-heating, and the resulting powder was stored under vacuum at −20 °C until use.

*Citrus × aurantium* L. fruits (bitter orange) were not subjected to any agronomic treatment and collected at full maturity in the Charterhouse of Pisa (Calci, Pisa, Italy). The albedo fraction was manually separated from flavedo (average thickness of periderm layer: 2 mm) using a ceramic blade to prevent the onset of oxidative processes and then sliced, freeze-dried and ground. The resulting powder was stored under vacuum at −20 °C until use.

Weak wheat flour type 0, lyophilized purple potatoes, and lyophilized albedo were analysed as previously reported [35].

### 2.3. Bread Preparation

Breadmaking tests were conducted at the Food Technology laboratory of the DAFE and each test formulation was carried out in triplicate.

The maintenance of sourdough was performed through consecutive back slopping in order to preserve the sourdough’s acidifying and leavening performances. Starter dough maintenance, back slopping, and baking were carried out under controlled operating conditions (time and temperature) [9]. In particular, bread making was carried out from a pre-ferment leavening agent made by sourdough (S) or baker’s yeast (Y), according to the two-step method of “biga.” Sourdough biga and baker’s yeast biga preparations, as well as bread making, were carried out according to Taglieri et al. [36].

Three formulations of bread (B) were produced using S biga (water 33%; sourdough 11%, strong wheat flour 56%) and/or Y biga (water 31%; baker’s yeast 1%, strong wheat flour 68%) as leavening agents, replacing a portion of weak wheat flour with cooked purple potato flour (15%) and cooked purple potato flour + lyophilized albedo (15% + 1%), as described in Table 1.

In particular, according to the results of preliminary trials (data not shown), breads containing more than the selected percentages of cooked purple potato flour and of *Citrus* albedo were evaluated not acceptable from a sensory point of view.

### 2.4. Chemical Composition of Bread

The moisture content of bread crumbs, taken from the centre of each slice, was determined on approximately 5 g sample drying at 105 °C until constant weight [37]. The pH value was measured following the AACC (American Association of Cereal Chemists) standard method [38]. A HygroPalm HP23-AW-A equipment (Rotronic AG, Bassersdorf, Switzerland) determined the water activity, while total titratable acidity (TTA) was measured as reported by Gélinas et al. [39]. The content of the main fermentative metabolites was determined by means of enzymatic kits (Megazyme Ltd., Wicklow, Ireland), [40,41,42,43].

### 2.5. Colour Parameters

The colour of the baked crumb samples was measured, after cooling, according to the CIE L*a*b* colour System by means of a tristimulus colorimeter (Eoptis, Mod. CLM-196 Benchtop, Trento, Italy). The analysis was performed on two centre slices of the loaf, on a crumb area of approximately 24 cm^2^ for each measure. The colour was defined on the base of the chromatic coordinates, lightness (L*), green-red (a*) and blue-yellow components (b*). The Chroma value C*, which is an expression of colour saturation, and hue value H*, which represents the tonality, were also used to evaluate the colour, and they were calculated, respectively, by the relations:

(1) $$C^{*}=\sqrt{a^{*}{}^{2}+b^{*}{}^{2}}$$

(2) $$H^{*}=\tan^{-1}\left(\frac{b^{*}}{a^{*}}\right)$$

The colour difference among samples was expressed as $\;\Delta E_{\mathrm{ab}}^{*}$:

(3) $$\Delta E_{\mathrm{ab}}^{*}=\sqrt{\Delta L^{*}{}^{2}+\Delta a^{*}{}^{2}+\Delta b^{*}{}^{2}}$$

### 2.6. Phenolic Compounds and Antioxidant Properties of Breads

#### 2.6.1. Extract Preparation

80% methanol and 5 g of bread were mixed in a ratio 1:4 (solid/liquid extraction, *w*/*v*) and sonicated for 30 min. The mixture was centrifuged (15 min, 1315× *g*) and the supernatant was filtered (0.45 μm) and stored at −20 °C until total phenol content evaluation.

As for the anthocyanin content, lyophilized purple potatoes, fresh sourdough and baker’s yeast breads (1.0 g) were extracted each in 6 mL of 2% HCl methanolic solution for 1 h [44]. The extracts were centrifugated (10 min, 1717× *g*) and the supernatants were analysed by ultra-high-performance liquid chromatography (UHPLC) coupled with an electrospray ionization source-high resolution-mass spectrometer (ESI-HR-MS).

#### 2.6.2. UHPLC-DAD-HR-ESI-MS/MS Analysis

The chemical characterization of the hydroalcoholic extracts of all bread samples was carried out by UHPLC-DAD-HR-MS (Vanquish Flex Binary pump LC system equipped with a Q Exactive Plus MS, Orbitrap-based FT-MS system; Thermo Fischer Scientific Inc., Bremem, Germany). Each sample (5 µL) was injected on a C-18 Kinetex^®^ Biphenyl column (100 × 2.1 mm, 2.6 μm particle size) equipped with a Security Guard TM Ultra Cartridge (Phenomenex, Bologna, Italy). Elutions were performed utilising metanoic acid in methanol 0.1% *v/v* (solvent A) and metanoic acid in water 0.1% *v/v* (solvent B) (linear solvent gradient: 5–55% A within 16 min; flow rate: 0.5 mL/min). For the anthocyanin extracts, metanoic acid in acetonitrile 0.1% *v/v* (solvent A) and metanoic acid in water 0.1% *v/v* (solvent B) were used (linear solvent gradient: 5 to 35% A within 4.5 min; flow rate: 0.5 mL/min). The temperatures of autosampler and column were 4 and 35 °C, respectively. UV spectra were measured at 254, 280 and 325 nm for phenols, and 520 nm for anthocyanins as preferential channels.

HR mass spectra were acquired in a scan range of *m*/*z* 150–1200 (*m*/*z* 770–1200 for the anthocyanin detection) in both ESI positive (useful for the detection of sugars, amino acids, anthocyanins, and glycoalkaloids) and negative ion mode (useful for the detection of organic acids, phenolic acids, and flavonoids), by using ionization parameters as previously reported [45].

All detected substances were tentatively identified on the basis of full mass spectra and fragmentation patterns according to literature data [46].

#### 2.6.3. Total Phenol Evaluation

The total phenol content of the methanolic extracts was determined spectrophotometrically at 700 nm according to the Folin–Ciocalteu method, as reported in a previous paper [47].

#### 2.6.4. Total Anthocyanin Evaluation

Anthocyanin levels were estimated on methanolic extracts as cyanidin-3-*O*-glycoside equivalents per gram of sample (dry matter, dm) as described by Sgherri et al. [22].

#### 2.6.5. Determination of Anti-Radical Activity

Both DPPH free radical method [48] and ABTS [49] were used, expressing the results as µmol Trolox equivalents (TE/g sample), in comparison with a calibration curve of Trolox (DPPH assay: 0–200 µmol L^−1^; ABTS assay: 0.2–1.5 mM).

### 2.7. Volatile Organic Compound (VOCs) Profile of Bread

Bread samples were characterized in terms of VOCs considering separately whole loaf (w) and sliced one (s), the latter without crust. The aromatic profile of bread samples was determined following the protocol previously described by Sanmartin et al. [35], using a SPME Supelco (50/30 μm coating thickness, St. Louis, MO, USA) for the adsorption of the volatile analytes, followed by gas chromatography-electron impact mass spectrometry (GC-EIMS) (Agilent Technologies Inc., Santa Clara, CA, USA).

### 2.8. Sensory Characterization of Bread (Crust and Crumb)

The “expert panel” of the DAFE, constituted by 10 assessors (4 males and 6 females, 23–60 years), selected and trained as described by Tonacci et al. [50] performed the sensory analysis, following the procedure described by Taglieri et al. [36]. The research obtained the approval of the ethical committee of the University of Pisa (Comitato Bioetico dell’Università di Pisa, protocol n. 0088081/2020).

### 2.9. Bread Shelf-Life Assessment

After baking, bread loaves were cooled for 2 h at room temperature, then sliced with an automatic slicing machine to 20 mm thickness.

Two slices were taken from the centre of each bread and stored at 23 °C under air, in paper bags closed in a three-layer bag (two plastic layers, outer nylon layer) (Food saver, Moncalieri (Torino, Italy) with an industrial packing machine (Lavezzini 450 GAS, Fiorenzuola d’Arda (PC), Italy). The sliced samples were inspected daily for water activity, moisture content, colour and compressibility. The shelf life of each bread was defined as the period before first mould appearance.

The staling rates of bread samples were determined during storage by measuring the compressibility with a penetrometer PNR-12 (Anton Paar, Rivoli (TO), Italy), according to the method described by Al Omari et al. [51] with some modifications. Briefly, each sample was compressed in five spots by a weight of 90 g for 10 s. The compression spots were identified by holes, on the four corners and in the centre, on a cardboard template, placed on the surface of each sample. The results were expressed as mm of penetration (0.1 mm = 1 penetration unit).

### 2.10. Statistical Analysis

All measurements are reported as a mean of three replicates. Two-way ANOVA (CoStat, Cohort 6.0) followed by the Tukey’s HSD test was performed on compositional data, with leavening agent and kind of fortification as main factors; significant differences were determined at different *p* values, with 0.05 selected as first level of significance.

VOCs profile were statistically analysed according to Sanmartin et al. [35] by means of JMP software package (SAS Institute, Cary, NC, USA), while a two-way ANOVA, choosing samples and panellists as main factors, after processing the results by Big Sensory Soft 2.0 (ver. 2018) was applied for sensory data [52].

Shelf-life results were processed by a C++ program based on a Root framework created by CERN in the European Laboratory of Particle Physics [53]. The program used a χ^2^ minimum model that allowed the following function to be reduced to a minimum (Equation (4)):

(4) $$\chi^{2}=\overset{N}{\underset{i=1}{\sum }}\frac{{\left(O_{i}-E_{i}\right)}^{2}}{\sigma_{i}^{2}}$$

where O_i_ is the i^-th^ measurement, E_i_ is the predicted value of the measurement and σ_i_ is the uncertainty of the measurement.

## 3. Results

### 3.1. Physical-Chemicals Parameters

#### 3.1.1. Main Ingredients’ Characterization

As reported in Table 2, the content of total phenols and anthocyanins showed significant differences among weak wheat flour, lyophilized purple potatoes and lyophilized albedo, in accordance with the antiradical activity detected.

The physical-chemical characterization of sourdough and baker’s yeast biga (Table 3) showed statistically significant differences for all the analysed parameter consistent with the different leavening agent used [54]. In particular, sourdough biga had a higher acidity due to the metabolic activity of the lactic acid bacteria (production of organic acids).

#### 3.1.2. Cooked Bread

Consistently, the main fermentative metabolite concentration in cooked breads were significantly affected by the leavening agent (Table 4). The addition of purple potatoes positively affected both lactic acid bacteria (LAB) and yeast metabolism, probably due to the higher sugar content of the purple potato flour compared to the control bread [19], with no further improvement detected when albedo was included in the recipe. A significant interaction between leavening agent and fortification factors was observed.

**Figure 1:**
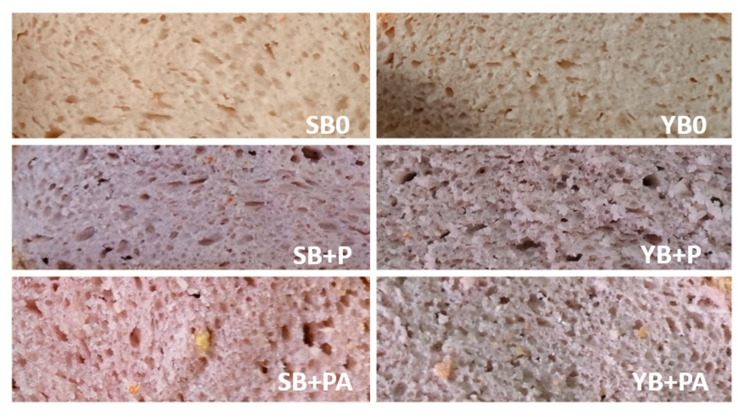
Pictures of the crumb of sourdough bread and baker’s yeast bread fortified with purple potatoes and albedo.

As reported in Table 5, the addition of purple potato and albedo determined a significant change in the colour of the bread samples (Figure 1), identified by different values of H* and C*.

The results show a significant effect of both fortification and the used leavening agent for all the parameters considered (Table 5).

Regardless the utilized leavening agent, the colour of crumb appeared significantly affected by fortification, in terms of both potatoes and albedo addition. The purple colour of the anthocyanin pigments derived from potatoes [19,55,56] induced a significant increase in darkness, and a change from white to light purple, highlighted by the increase in redness and the decrease in yellowness. Hue and Chroma significantly changed as a consequence of the albedo addition.

Furthermore, the Chroma of the crumb significantly changed depending on the leavening system. Indeed, with the same flour composition, sourdough bread appeared more vibrantly colourful when compared to baker’s yeast one.

According to the above-discussed results, all bread samples could be visibly discriminated in colour when compared to each other, as outlined by the distance between the chromatic coordinates ($\Delta E_{\mathrm{ab}}^{*}$) (Table 6).

### 3.2. Phenolic Compounds and Antioxidant Properties of Breads

The replacement of part of the wheat flour with purple potato and albedo determined a significant enhancement of the phytochemical profile of fortified breads, in accordance with their higher content of total phenols and anthocyanins (Table 2). Total phenols were increased by at least 80% by fortification with purple potato flour because of its anthocyanin content. This value was further enhanced following albedo addition (Table 7). The anti-radical activity doubled when the purple potato flour was added and even triplicated in the presence of *Citrus* albedo. In particular, the leavening agent had a significant influence, as SBs showed higher levels of total phenols and anti-radical activity than BY ones (Table 7). These results are consistent with previous studies [57,58,59] showing how the enzymatic activity of sourdough lactic acid bacteria could hydrolyse the complex phenolics and their glycosylated forms into phenolic acids. Leavening agent and fortification factors exhibited a significant interaction (*p* < 0.05) for all the parameters considered, with the exception of the total anthocyanins.

**Figure 2:**
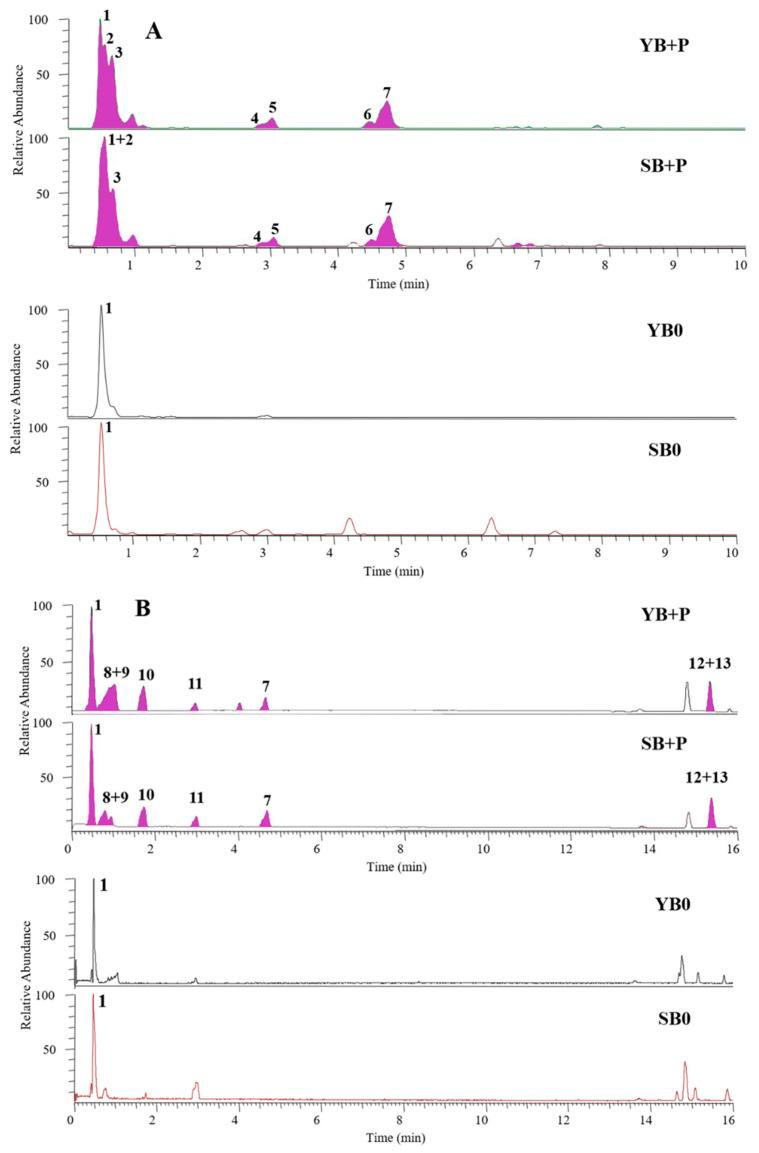
Negative (panel (**A**)) and positive (panel (**B**)) UHPLC-HR-ESI-MS profiles of hydroalcoholic extracts from SB and YB enriched with purple potatoes (P), compared to SB0 and YB0 (controls). For peak identification, see Table S1 in Supplementary material.

The chemical profiles of all hydroalcoholic extracts of bread samples were analysed by UHPLC-DAD-HR-ESI-MS. In particular, the attention was focused on secondary metabolites of both purple potato and sour orange albedo, that were preserved in all bread samples during the whole process of preparation. Potatoes are known to be rich in phenolic acids and glycoalkaloids [60], while pigmented potato varieties also contain a high content of anthocyanins [61]. The HR-LC-MS profiles of baker’s yeast and sourdough breads enriched with purple potatoes were registered in negative ion mode for phenolic acids (Figure 2A) and in positive ion mode for glycoalkaloids (Figure 2B). The chromatogram in Figure 2A showed, in addition to saccharose (compound **1**) and citric acid in two isomeric forms (compounds **2** and **3**), the main phenolic acids detected in bread samples enriched with purple potatoes, tentatively identified as three caffeoylquinic acid isomers (compounds **4**, **5** and **7**) and caffeic acid (compound **6**). The spectral data of the identified compounds are shown in Table S1 of Supplementary material and are in agreement with the literature data [62]. All three caffeoylquinic acid isomers showed the same deprotonated molecule [M-H]^−^ at *m/z* 353.0877 and the same ion base peak at *m/z* 191.05 in MS/MS experiments due to the loss of a caffeoyl residue; thus, it is not possible to establish the exact position on the quinolyl moiety and discriminate among chlorogenic acid (3-*O*-caffeoylquinic acid), cryptochlorogenic acid (4-*O*-caffeoylquinic acid), and neochlorogenic acid (5-*O*-caffeoylquinic acid). Since chlorogenic acid is the main caffeoylquinic acid reported in potatoes [63] it can be assumed that it is represented by compound **7**.

In the chromatogram shown in Figure 2B, it can be observed that glycoalkaloids were also present in the bread enriched with purple potatoes; in particular, α-chaconine (compound **12**, [M + H]^+^ at *m/z* 852.5081) and α-solanine (compound **13**, [M + H]^+^ at *m/z* 868.5036) showed coelution in the chromatographic process, in agreement with results reported by Ieri et al. [62]. As observed in Figure 2, bread plus purple potatoes was also enriched in the free amino acids leucine/isoleucine (**8**), tyrosine (**9**), phenylalanine (**10**), and tryptophan (**11**) coming from tubers.

**Figure 3:**
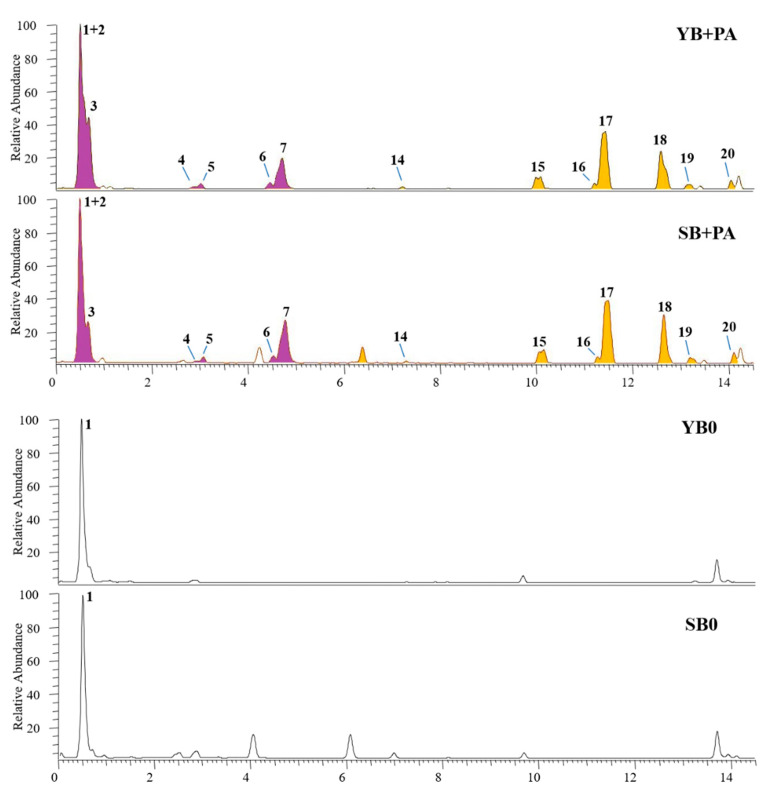
UHPLC-HR-ESI-MS profiles of hydroalcoholic extracts from SB and YB enriched with purple potatoes and *Citrus x aurantium* albedo, compared to SB0 and YB0 (controls). Compounds from albedo and potatoes were represented by yellow and purple peaks, respectively. For peak data, see Table S2 in Supplementary material.

When *C. aurantium* albedo was added to both sourdough and baker’s yeast breads already enriched with purple potatoes, additional phenolic compounds were detected in the LC-MS profiles. As shown in Figure 3, yellow peaks were attributed to albedo constituents. According to the literature data [64,65], the major components were represented by the flavone *C*-glucoside vicenin-2 (peak **14**), a flavone *O*-glucoside tentatively identified as apigenin 7-*O*-rutinoside or 7-*O*-neohesperidoside (peak **16**), three flavanone *O*-glycosides (peaks **15**, **17** and **18**) represented by eriocitrin, naringenin and neohesperidin, and 3-hydroxy-3 methyl-glutaryl flavanone *O*-glycosides such as melitidin (peak **19**) and brutiedirin (peak **20**). Flavonoids are very common in fruits of the *Citrus* genus and are very interesting for their wide biological activity [66]. At last, LC-MS analyses in positive ion mode (data not shown) confirmed the presence in both SB+P and YB+P samples of the two glycolkaloids **12** and **13** from purple potatoes.

**Figure 4:**
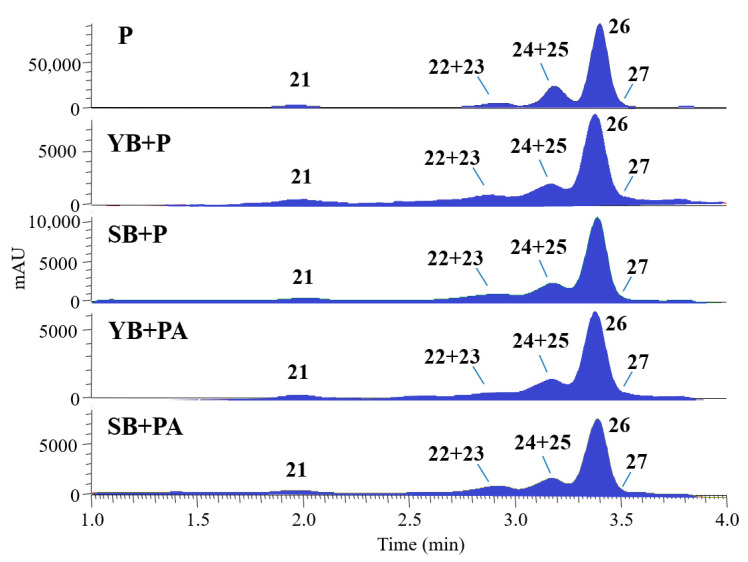
UHPLC-DAD profiles registered at 520 nm of anthocyanin extracts from SB and YB enriched with purple potatoes (P) and *Citrus x aurantium* albedo (A). For peak identification, see Table S3 in Supplementary material.

The anthocyanin content of all bread samples enriched with purple potatoes was investigated by UHPLC-DAD-HR-ESI-MS analyses in positive ion mode and at 520 nm on methanol extracts acidified with 2% HCl to carry out a selective extraction of these molecules. Results showed the presence of seven anthocyanins (peaks **21**–**27**, Figure 4), malvidin and petunidin derivatives, identified as follows: malvidin 3-*O*-rutinoside-5-*O*-glucoside (**21**), malvidin 3-*O*-caffeoyl-rutinoside-5-*O*-glucoside (**22**), petunidin 3-*O*-caffeoyl-rutinoside-5-*O*-glucoside (**23**), petunidin 3-*O*-feruloyl-rutinoside-5-*O*-glucoside (**24**), petunidin 3-*O*-*p*-coumaroyl-rutinoside-5-*O*-glucoside (**25**), malvidin 3-*O*-*p*-coumaroyl-rutinoside-5-*O*-glucoside (**26**), and malvidin 3-*O*-feruloyl-rutinoside-5-*O*-glucoside (**27**). Malvidin 3-*O*-*p*-coumaroyl-rutinoside-5-*O*-glucoside (**26**) was the most abundant, in agreement with the literature [61,62] (Figure 4).

Compared to raw materials, bread samples fortified with potato purples showed the same chemical profiles, while in bread samples fortified with sour orange albedo some compounds were lost during the bread preparation, such as caffeic acid glucoside, feruloyl acid glucoside, diosmetin 6,8-di-*C*-glucoside, naringin glucoside, diosmetin 8-*C*-glucoside/6-*C*-glucoside, and 3-hydroxy-3-methyl-glutaryl-neoericitrin/eriocitrin hexoside. The UHPLC-MS profiles of purple potatoes and sour orange albedo are shown in Figure S1 (Supplementary materials), while the compounds detected in the *Citrus aurantium* albedo, but not found in bread samples enriched with albedo are listed in Table S5 (Supplementary materials) together with MS data.

### 3.3. Volatile Bouquet in the Headspace Emissions of Whole and Sliced Breads

The 97.0–99.6% (50 compounds) of the total VOCs spontaneously emitted (Table S4 in the Supplementary material) was identified.

Four main chemical classes of volatiles were identified, among which non-terpene derivatives clearly prevailed (78.5–97.7%). Nitrogen derivatives, pyrroles, and pyrazines were only emitted when purple potatoes and/or albedo were added. Non-terpene derivatives were further divided into chemical subclasses, as a consequence of their high percentages. The emission of all SB samples was characterized by the presence of organic acids like acetic acid, especially when potatoes or albedo were absent, while non-terpene alcohol/ethers were mainly present in YB emissions. In the case of non-terpene esters, a marked emission was observed only for sourdough bread when no other ingredient was added to the dough. Indeed, the other samples showed comparable emission patterns for this chemical class.

All samples, particularly those prepared with sourdough and when the other ingredients were added, were characterised by non-terpene aldehydes/ketones; conversely non-terpene hydrocarbons were always detected in smaller percentages.

**Figure 5:**
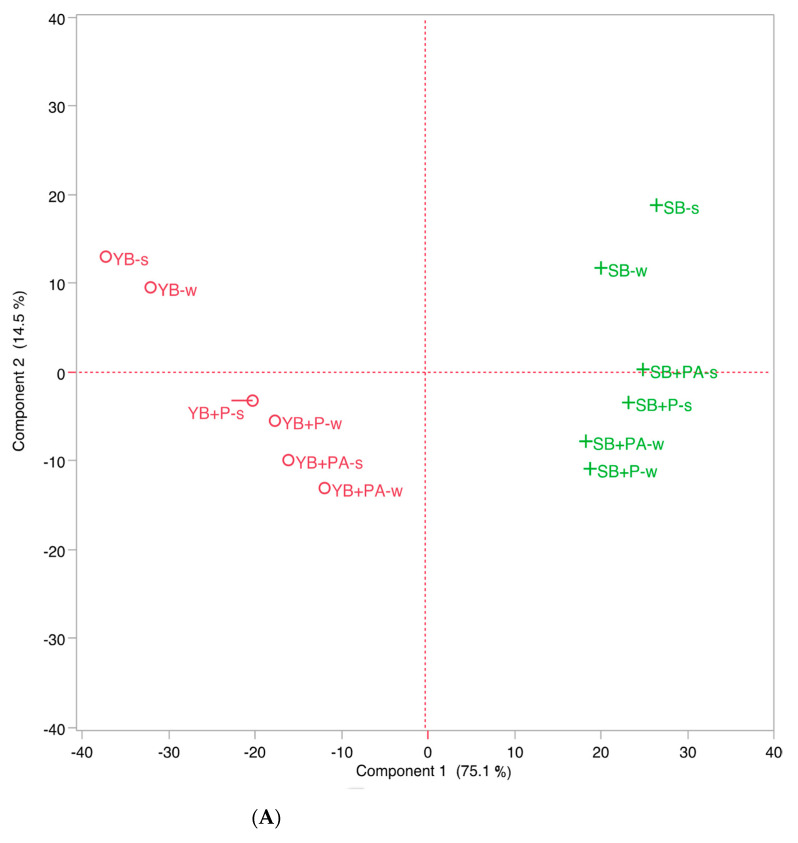

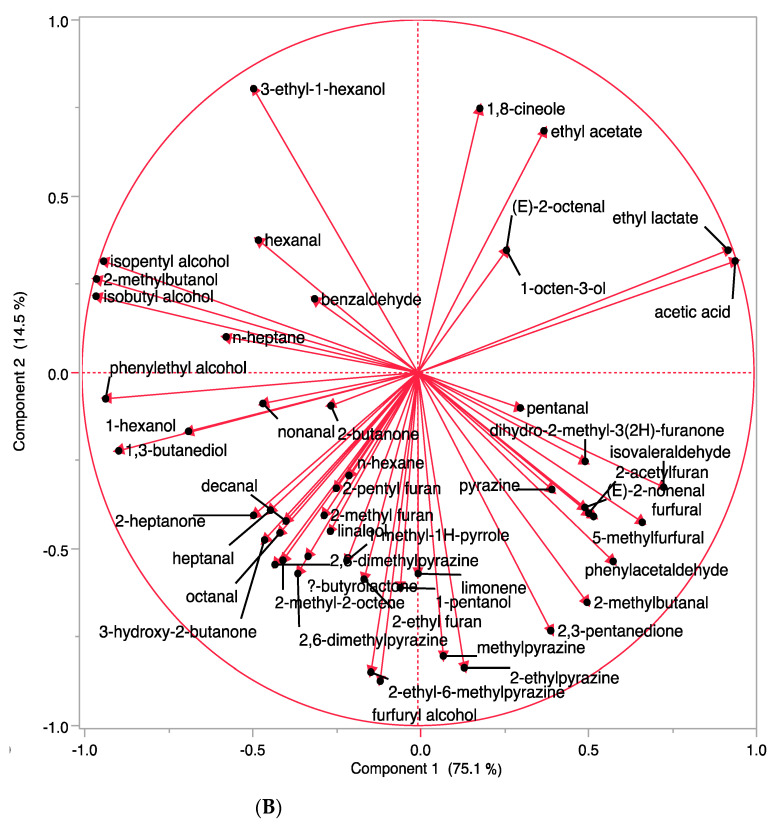
(**A**) Scores and loading plots (**B**) of PCA of bread VOCs (w = whole or s = sliced without crust).

The PCA score plot (total explained variance 89.6%) (Figure 5A,B) clearly shows that, regardless the considered fraction (crust and crumb) the control breads (SB0 and YB0) are significantly different, due to the leavening system, indicating the strong influence of the type of leavening agent on the aroma.

Regardless the considered fraction (crust and crumb), the samples could be further grouped according to the other ingredients added to the dough, as a function of the formulation. All the SBs showed positive loadings on PC1, mainly because of the presence of acetic acid, ethyl acetate, ethyl lactate and 1,8-cineole. Among them, the addition of the other ingredients is clearly evidenced by the negative loadings on PC2 with regard to the positive ones observed for SB0 and YB0 for the increased emission of furfural, 2-methylbutanal, isovaleraldehyde and limonene. YB0 had negative PC1 and positive PC2 loadings due to the presence of isopentyl alcohol, isobutanol and hexanal in the emission profile, while the addition of the other ingredients, similarly to the previous samples, was again highlighted by the negative loadings on PC2 due to the appearance of 2-pentylfuran, furfuryl alcohol and pyrazines (Figure 5B).

### 3.4. Sensorial Parameters

Bread sensorial profiles were strongly affected by the formulation, indeed samples significantly differed for most of the parameters evaluated by the panelists (Table 8).

Visual evaluation of the colour change was in accordance with the results of instrumental measurement. Moreover, the crumb smell profile was more influenced by fortification than the leavening agent, as the presence of potato flour seemed to be predominant, determining a lower smell intensity and a reduced perception of wheat hints. As expected, a higher perception of acetic smell was detected in SB samples.

With respect to taste, a higher acidity was detected in both crumb and crust in SB samples; this perception increased when albedo was also added. A salted taste was perceived especially in sourdough breads, both crumb and crust fraction, although no salt was added at all. This was probably due to the combined effect of acidification and proteolysis promoted by LAB activity [67], particularly enhanced in sourdough bread with albedo.

Moreover, the panellists perceived a higher bitterness in the presence of albedo regardless of the leavening system used, probably due to *Citrus* high phenolic compound content. A significant and unpleasant aftertaste was detected in sourdough bread fortified with albedo probably due to the synergy between the acidity and bitter taste.

Considering rheological features, the main differences highlighted by the panellists were related to the fermentative activity of the leavening agent. In particular, the alveoli of SB had higher dimensions than YB, regardless of the kind of fortification. However, the change of flour composition due to the addition of potato and albedo increased the compactness of the crumb, reducing the presence of rips in the bread structure, irrespective of the leavening agent (Table 8).

**Figure 6:**
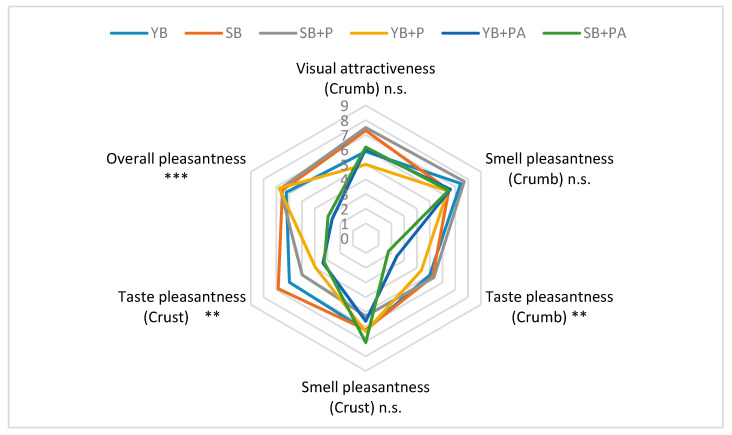
Hedonic profile of cooked breads (for both crumb and crust). Significance level *** *p* < 0.001; ** *p* < 0.01; ns: not significant (*p* > 0.05).

Preliminary data about the sensory acceptance of the considered formulation were finally collected by the evaluation of some hedonic parameters (Hedonic indices): visual attractiveness, taste and smell pleasantness, and overall pleasantness (Figure 6). According to the hedonic profile, the addition of albedo to both SB and YB induced the worst taste perception, due to their high bitterness and acidity, resulting in the lowest values of overall pleasantness (Figure 6).

Moreover, according to Taglieri et al. [36], in order to evaluate the potential acceptance of the new formulations, the overall hedonic index of each bread was calculated, starting from the mean of the hedonic indices converted on a scale from 0 to 10, according to the following equation:

(5) Overall hedonic index= MEAN [Hedonic indices]∗1,11 

**Figure 7:**
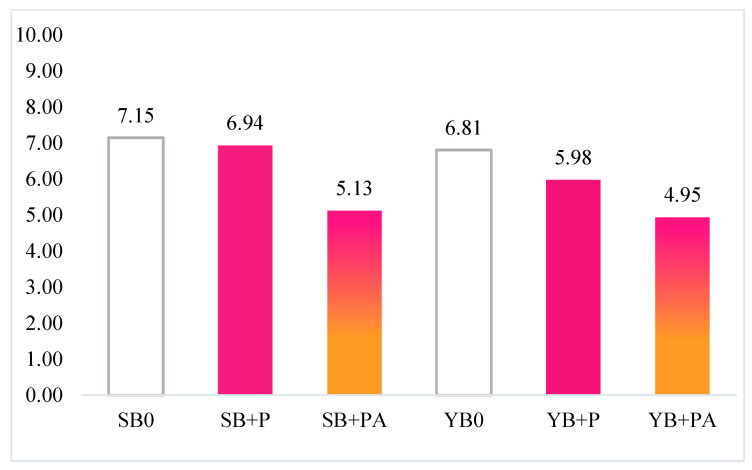
Overall hedonic indexes of breads.

As reported in Figure 7, the fortification with purple potato was positively evaluated, especially when sourdough was used. As expected, the addition of albedo negatively affected the hedonic perception, determining a worsening of the whole acceptance (~−30%) regardless the leavening agent used (Figure 7).

### 3.5. Shelf Life: Preliminary Results

**Figure 8:**
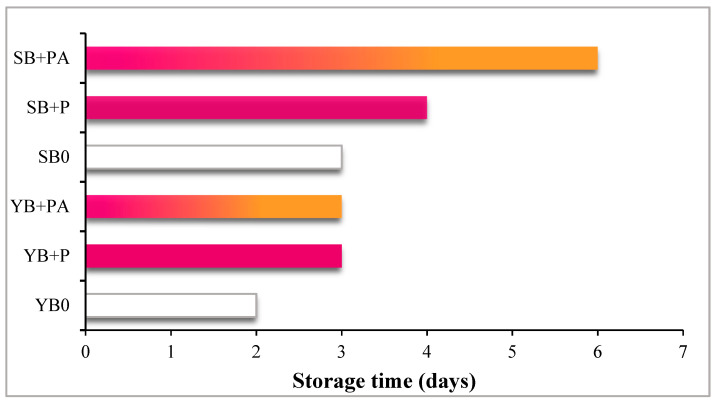
Period of time (days) of storage before mould appearance on cooked bread.

The appearance of mould on the surface of the bread slices was selected as a parameter to define the limit of acceptability of bread during storage. The bread slice samples were observed daily for mould detection and for each formulation the shelf-life evaluation ended when the first evident moulds appeared. Bread shelf life was affected by both the leavening agent and the fortification used. As shown in Figure 8, when baker’s yeast was used as the leavening agent, the first obvious spoilage was noticed after 2 days on the YB0 sample, while fortification (YB+P and YB+PA samples) prolonged by one day bread shelf life. Regarding sourdough, the first mould appeared after 3 days on SB0 and bread shelf life was extended by one day more by fortification with purple potatoes (SB+P), being even doubled by the addition of both purple potatoes and *Citrus* albedo (SB+PA) (Figure 8).

The length of time before the appearance of mould was significantly different for each bread formulation, although the changes in a_w_ were not so significant as to justify this trend (Table 9).

At the end of the bread shelf life (t = t_fin_), differences in colour were also detected for each sample (Table 9). In particular, the colour of the fortified bread was more affected by storage time, while both the control samples showed extremely small differences in comparison with time 0 (YB ${\Delta E}_{\mathrm{ab}\;}^{*}=0.2$; SB0 $\Delta E_{\mathrm{ab}\;}^{*}=1.4$): YB+P showed a higher change (${\Delta E}_{\mathrm{ab}\;}^{*}=6.5)$ than the analogous sample produced with sourdough (${\Delta E}_{\mathrm{ab}\;}^{*}=3.7)$, while the addition of albedo counteracted the colour modification (YB+PA ${\Delta E}_{\mathrm{ab}\;}^{*}=3.4$; SB+PA ${\Delta E}_{\mathrm{ab}\;}^{*}=3.0$). We observed a reduction in L* parameter, linked to an increased opacity due to the migration of moisture from crumb to crust, especially for YB+P. Regarding sourdough samples, the change in colour was due not only to L* parameter, but also to a slight reduction in both red and blue components. The colour change from t = t_0_ could be therefore explained also by the oxidation of anthocyanins, especially evident in the samples where the red colour linked to the presence of anthocyanins was enhanced by the lower pH.

Bread shelf-life is inevitably associated with bread staling and, therefore, with the loss of water and the deterioration of the structural properties of the bread crumb. In this context, the texture properties (compressibility), as well as the weight decrease, as a measure of the water loss were monitored daily during storage. It should be stressed that we observed only the first phase of the bread staling process due to its slower kinetics if compared to the appearance of the first mould, that defined the acceptability limit and, consequently, the end of the trial (t = t_fin_).

**Figure 9:**
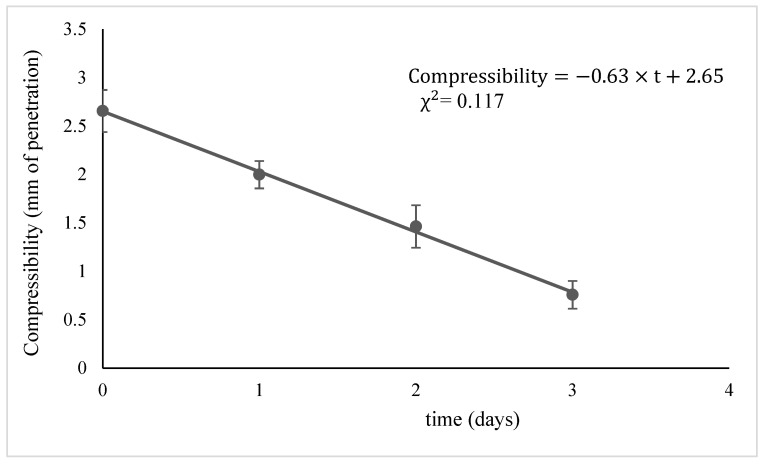
Decrease in compressibility (mm of penetration) of sample YB+PA during storage time.

In this first phase, the kinetics of the loss of compressibility could be described by a zero-order kinetics, following a linear trend, as shown in Figure 9 for the sample YB+PA:

The trend of compressibility as a function of time could be therefore described by the following equation (Equation (6)):

(6) $${\;\mathrm{Compressibility}}_{t=t}=k_{c}\times t+q_{c}$$

where

k_c_ = kinetic constant (mm·days^−1^)
t = time (days)
q_c_ = ${\mathrm{Compressibility}}_{t=0}$(mm)

A similar approach was used to describe the weight decrease (%), which trend followed an analogous zero order kinetics, according to the following linear equation (Equation (7)) where the q_w_ parameter was zero (${\mathrm{weight}\;\mathrm{decrease}}_{t=0}=0)$:

(7) $${\;\mathrm{Weight}\;\mathrm{decrease}}_{t=t}=k_{w}\times t$$

k_w_ = kinetic constant (days^−1^)
t = time (days).

The values of the kinetic parameters, together with the chi-square ($\chi^{2}$), calculated for each bread sample, are reported in Table 10.

According to the results, it was possible to observe a higher value of k associated to a faster loss of compressibility of bread, when baker’s yeast was used as the leavening agent. At the same time, fortification increased the compressibility of bread, irrespective of the leavening agent, although the addition of albedo induced a lower penetrability.

Accordingly, the use of baker’s yeast determined a faster decrease of bread weight during time. Moreover, the fortification did not differently affect sourdough bread weight loss, while it was possible to observe a slower water loss with the fortification of baker’s yeast bread, especially when albedo was also added.

## 4. Discussion

Bakery products traditionally play an important role worldwide in human diet and different strategies for the development of functional bread with enhanced contents of natural bioactive compounds have been proposed in recent studies [35,68,69].

In this context, red and purple coloured flesh potatoes, as well as by-products from *Citrus* fruit processing, are promising sources of bioactive compounds for the production of novel foods.

Indeed, red and purple potatoes contain large quantities of phenolic acids (chlorogenic, neochlorogenic and cryptochlorogenic, gallic, ferulic, protocatechuic, caffeic, cynnamic, synapic and p-coumaric acids), flavonoids (catechin, epicatechin, kaempferol, naringenin, rutin), and the heat-stable anthocyanins [70], which beneficial functions such as scavenging of free radicals, anti-mutagenicity, anticarcinogen activity and anti-hypertensive effects are widely described in the literature [71,72].

Our results confirmed that secondary metabolites from Vitelotte potatoes were preserved during baking, resulting in a significant improvement of the phenolic content and therefore the antioxidant status of fortified breads. Moreover, as previously reported [36,67], the leavening agent affected the levels of health-promoting compounds, with an improvement of phenols, flavonoids, and TEAC in sourdough breads. According to the literature [73], chlorogenic acid was the predominant phenolic acid deriving from purple potatoes. Together with its protective effect against reactive oxygen species (ROS) [74], this compound was found to promote the growth of Bifidobacterium species that could be beneficial for gut health [75].

Nevertheless, to verify if the cooked breads developed in the current research have a real pro-health effect, clinical studies of fortified breads should be carried out to evaluate the bioavailability of the added bioactive compounds. The interaction of phenols with proteins and the consequent formation of polyphenol-protein complexes could indeed affect their digestibility in the gastrointestinal tract and therefore their bioavailability [76,77].

Our results on fortified breads with cooked purple potato flour, consistently showed the presence of amino acids such as isoleucine, tyrosine, phenylalanine, and tryptophan, that positively affected the nutritional value of the product as well as its aromatic profile due to the Maillard reaction. Recent studies pointed out the high quality of potato proteins compared to other plant-based and the similarity to animal-based proteins in terms of essential amino acid content [78]. According to Pęksa et al. [79,80], purple fleshed Vitelotte potatoes are rich in the essential amino acids threonine, cystine, and isoleucine and also in proline, glycine, and alanine when compared to other potatoes.

Glycoalkaloids such as α-chaconine and α-solanine were also found in bread fortified with purple potatoes. Indeed, potatoes contain glycoalkaloids which, in high concentrations, can be toxic to humans, but in low concentrations, can have beneficial effects, such as the inhibition of the growth of cancer cells [81,82,83]. According to the German Federal Institute for Risk Assessment, the total glycoalkaloid content of potatoes should not be higher than 100 mg/kg fresh weight to avoid the exceedance of No Observed Adverse Effect Level NOAEL (0.5 mg/kg bw per day) [84]. The average amount found in commercial potato flesh is generally less than 100 mg/kg fresh weight [84,85] and coloured-flesh potatoes exhibit lower levels compared to yellow-fleshed varieties [86]. The evaluation of the antinutritional compounds under different operating conditions is currently ongoing and the results will be the subject of a future publication.

The addition of *C. aurantium* albedo further improved the phenolic profile of fortified breads due to the enrichment with flavonoids such as naringin and neohesperidin exhibiting a number of beneficial properties, including antioxidant, anti-inflammatory, neuroprotective, anticancer, immunomodulatory, and antidiabetic properties [29,87]. However, although albedo conferred the best antioxidant profile to breads, its addition negatively affected bread sensory profile, determining a higher perception of aftertaste and bitterness, probably connected with the high polyphenol content of the albedo [88,89,90,91], irrespective of the leavening agent used.

Bread fortified with purple potatoes and albedo exhibited not only a higher phenolic content and antioxidant capacity, but also a longer shelf life, especially when the sourdough was used as leavening agent [6]. Albedo is, indeed, a good source of fibre and pectin [33,91] that could positively influence bread shelf life. Previous studies have shown that the partial replacement of wheat flour with dried fruit peel powder [28,92] determined a higher ability to bind large amounts of water [93]. The hydrophilic characteristics of gluten in flour can be enhanced during bread making by the addition of pectins and polyphenols as well by the formation of H bonds with water, polyphenols and polysaccharides [33]. Moreover, Han et al. demonstrated that when the addition of orange peel powder was no greater than 5%, a significant inhibition in the retrogradation of wheat dough was detected, while an opposite tendency was observed with higher percentages [94]. However, changes in the mechanism of staling, as well as structure modification as a consequence of fortification, should be further investigated.

## 5. Conclusions

To sum up, bread fortified with purple potatoes and albedo exhibited a higher content of phenols and a longer shelf life than control breads; this trend was further improved by the use of sourdough as the leavening system. Moreover, both the fortification and the leavening system deeply affected the sensory expression and the VOCs emission of the proposed breads. In the present experimental condition, however, bread formulation needs further optimization, in terms of percentage of albedo addition, with the aim of improving the sensory acceptance of the final product.

Based on all the results obtained, the use of purple potatoes and *Citrus* albedo, if properly formulated, could represent a valuable strategy in bread making for the development of new high-quality products, with a longer shelf-life.

## Figures and Tables

**Table 1:** Codes of bread samples and related formulations.

	Weak Wheat Flour	Water	S Biga	Y Biga	Cooked Purple Potato Flour	*Citrus* Albedo
SB0	52%	32%	16%	-	-	-
SB+P	44%	32%	16%	-	8%	-
SB+PA	43.25%	32%	16%	-	8%	0.75%
YB0	52%	32%	-	16%	-	-
YB+P	4%	32%	-	16%	8%	-
YB+PA	43.25%	32%	-	16%	8%	0.75%

**Table 2:** Dry matter, total phenols, antiradical activity and total anthocyanins of weak wheat flour type 0, lyophilized purple potatoes and lyophilized albedo. Data presented are the mean of three replicates.

	*p*-Value ^1^	Weak Wheat Flour	Lyophilized Purple Potatoes (P)	Lyophilized Albedo (A)
Dry matter (% dm)	***	89.38 ^b^	95.31 ^a^	86.34 ^c^
Total phenols (mg GAE/g dm)	***	0.25 ^c^	5.38 ^b^	42.71 ^a^
Antiradical activity (µmol TE/g dm)	***	0.993 ^c^	51.448 ^b^	85.06 ^a^
TEAC (µmol TE/g dm)	***	0.504 ^c^	27.248 ^b^	43.637 ^a^
Total anthocyanins (mg cyanidin-3-*O*-glycoside/kg dm)	***	b.d.l	2263 ^a^	33 ^b^

Notes: ^1^ Significance level *** *p* < 0.001. In the same row, different letters indicate significant differences among samples. b.d.l. = below detection limit. GAE = gallic acid equivalents; TE = Trolox equivalents; TEAC = Trolox equivalent antioxidant capacity.

**Table 3:** Dry matter (dm %), pH, total titratable acidity (TTA) and main fermentative metabolites of sourdough and baker’s yeast biga.

	*p*-Value ^1^	Sourdough Biga	Baker’s Yeast Biga
Dry matter (% dm)	***	57.7 ^b^	61.8 ^a^
pH	***	3.84 ^b^	5.84 ^a^
Total titratable acidity (meq lactic acid/g dm)	***	0.102 ^a^	0.008 ^b^
Acetic acid (mmol/g dm)	*	0.043 ^a^	0.037 ^b^
d-Lactic acid (mmol/g dm)	***	0.026 ^a^	b.d.l ^2^
l-Lactic acid (mmol/g dm)	***	0.102 ^a^	b.d.l.
Ethanol (mmol/g dm)	**	0.113 ^b^	0.308 ^a^

Notes: ^1^ Significance level *** *p* < 0.001, ** *p* < 0.01, * *p* < 0.05. In the same row, different letters indicate significant differences among samples. ^2^ b.d.l: below detection limit.

**Table 4:** Water activity (a_w_), dry matter (dm %), pH, TTA and fermentative metabolites of cooked breads. Data presented are the mean of three replicates.

	*p*-Value ^1^	SB0	SB+P	SB+PA	YB0	YB+P	YB+PA
Water activity	n.s./§§§/¶¶	0.958 ^a^	0.955b	0.952 ^c^	0.958 ^a^	0.954 ^b^	0.953 ^c^
Dry matter (%)	n.s./***/n.s.	57.3	54.0	55.5	57.3	53.4	55.3
pH	***/§§§/¶¶¶	4.10 ^e^	4.61 ^c^	4.32 ^d^	5.86 ^b^	6.06 ^a^	5.78 ^b^
TTA (meq lactic acid/g dm)	***/§§§/¶¶¶	0.044 ^b^	0.043 ^b^	0.062 ^a^	0.006 ^d^	0.008 ^d^	0.012 ^c^
Acetic acid (mmol/g dm)	n.s./n.s./n.s.	0.061	0.045	0.043	0.044	0.041	0.050
d-Lactic acid (mmol/g dm)	***/§§§/¶¶¶	0.011 ^b^	0.019 ^a^	0.020 ^a^	b.d.l. ^2^	b.d.l.	b.d.l.
l-Lactic acid (mmol/g dm)	***/§§§/¶¶¶	0.067 ^b^	0.079 ^a^	0.083 ^a^	b.d.l.	b.d.l.	b.d.l.
Ethanol (mmol/g dm)	***/§/¶	0.032 ^c^	0.059 ^b^	0.051 ^b^	0.040 ^c^	0.084 ^a^	0.087 ^a^

Notes: ^1^ Significance level—***/§§§/¶¶¶: *p* < 0.001; ¶¶: *p* < 0.01; §/¶: *p* < 0.05; ns: not significant (*p* > 0.05). In the same row, different letters indicate significant differences among samples. Legend for symbol’s meaning: * = Leavening agent as main factor for ANOVA; § = fortification as main factor for ANOVA; ¶ = interaction among main factors (leavening agent) (fortification). ^2^ b.d.l: below detection limit.

**Table 5:** Colour parameters L*a*b* of the analysed bread.

	*p*-Value ^1^	SB0	SB+P	SB+PA	YB0	YB+P	YB+PA
L*	n.s./§§§/¶¶¶	66.62 ^a^	57.75 ^c^	56.46 ^c^	68.97 ^a^	60.64 ^b^	52.18 ^d^
a*	***/§§§/¶¶¶	0.69 ^c^	5.98 ^a^	5.66 ^a^	0.29 ^c^	3.53 ^b^	3.79 ^b^
b*	n.s./§§§/¶¶¶	15.92 ^a^	7.53 ^e^	11.28 ^c^	14.69 ^b^	8.60 ^d^	11.82 ^c^
H*	***/§§§/¶¶¶	1.53 ^a^	0.90 ^e^	1.11 ^d^	1.55 ^a^	1.18 ^c^	1.26 ^b^
C*	***/§§§/¶¶	15.94 ^a^	9.61 ^d^	12.62 ^c^	14.69 ^b^	9.30 ^d^	12.41 ^c^

Notes: ^1^ Significance level—***/§§§/¶¶¶: *p* < 0.001; ¶¶: *p* < 0.01; ns: not significant (*p* > 0.05). In the same row, different letters indicate significant differences among samples. Legend for symbol’s meaning: * = Leavening agent as main factor for ANOVA; § = fortification as main factor for ANOVA; ¶ = interaction among main factors (leavening agent) · (fortification).

**Table 6:** Cie L*a*b* colour differences ($\Delta E_{\mathrm{ab}}^{*})\;$ among cooked bread samples.

$\Delta E_{ab}^{*}$	SB0	SB+P	SB+PA	YB0	YB+P	YB+PA
SB0		13.30	12.22	2.67	9.87	15.32
SB+P			3.98	14.47	3.93	7.37
SB+PA				14.03	5.40	4.71
YB0					10.82	17.39
YB+P						9.05
YB+PA						

**Table 7:** Effect of leavening agent and fortification on total phenols, total anthocyanins, anti-radical activity of bread samples.

	*p*-Value ^1^	SB0	SB+P	SB+PA	YB0	YB+P	YB+PA
Total phenols (mg GAE/g dm)	***/§§§/¶	0.867 ^d^	1.571 ^b^	2.304 ^a^	0.589 ^e^	1.273 ^c^	1.684 ^b^
Total anthocyanins (mg cyanidin-3-*O*-glycoside/kg dm)	***/§§§/n.s.	b.d.l.	264	237	b.d.l.	230	217
DPPH (μmol TE/g dm)	***/§§/¶	1.521 ^d^	5.766 ^b^	6.583 ^a^	1.493 ^d^	4.835 ^c^	5.644 ^b^
TEAC (μmoL TE/g dm)	***/§§§/¶	0.843 ^d^	2.885 ^b^	3.195 ^a^	0.848 ^d^	2.498 ^c^	2.885 ^b^

Notes: ^1^ Significance level—***/§§§: *p* < 0.001; §§: *p* < 0.01; ¶: *p* < 0.05; ns: not significant (*p* > 0.05). In the same row, different letters indicate significant differences among samples. Legend for symbol’s meaning: * = Leavening agent as main factor for ANOVA; § = fortification as main factor for ANOVA; ¶ = interaction among main factors (leavening agent) ×(fortification). b.d.l. = below detection limit.

**Table 8:** Sensory profiles of breads evaluated by panellists.

Sensory Parameters	*p*-Value ^1^	SB0	SB+P	SB+PA	YB0	YB+P	YB+PA
Crumb								
Crumb colour intensity (White scale)	***	5.25 ^a^	0.00 ^b^	0.00 ^b^	4.48 ^a^	0.00 ^b^	0.00 ^b^
Crumb colour intensity (Purple scale)	***	0.00 ^b^	4.08 ^a^	4.30 ^a^	0.00 ^b^	2.28 ^ab^	2.88 ^ab^
Presence of rips	***	1.63 ^a^	0.58 ^b^	0.98 ^ab^	1.97 ^a^	0.43 ^b^	0.73 ^b^
Alveoli dimension	*	3.10 ^a^	3.13 ^a^	2.33 ^a^	1.95 ^b^	1.83 ^b^	1.13 ^b^
Homogeneity of alveolation	*	4.45 ^b^	4.50 ^b^	4.88 ^b^	5.18 ^ab^	6.02 ^a^	6.63 ^a^
Smell intensity	*	6.63 ^a^	4.70 ^ab^	4.60 ^ab^	4.58 ^ab^	3.20 ^b^	4.15 ^ab^
Wheat smell	*	3.38 ^a^	2.73 ^b^	3.05 ^ab^	4.38 ^a^	1.88 ^b^	2.38 ^b^
Yeast smell	n.s.	3.33	2.08	2.28	3.50	3.98	3.55
Acetic smell	***	4.48 ^a^	1.90 ^ab^	1.98 ^ab^	0.38 ^b^	0.30 ^b^	1.05 ^b^
Frankness	*	6.2 ^ab^	6.38 ^a^	5.20 ^b^	7.73 ^a^	6.75 ^a^	6.10 ^ab^
Salted taste	***	2.83 ^ab^	2.13 ^ab^	3.95 ^a^	1.10 ^b^	1.25 ^b^	1.05 ^b^
Acid taste	***	3.65 ^ab^	4.70 ^ab^	5.03 ^a^	0.20 ^c^	0.15 ^c^	1.68 ^bc^
Bitter taste	***	1.93 ^b^	0.68 ^b^	6.18 ^a^	1.20 ^b^	1.85 ^b^	5.53 ^a^
Aftertaste	*	1.43 ^ab^	1.38 ^ab^	3.98 ^a^	0.33 ^b^	2.25 ^ab^	3.15 ^ab^
Springiness	n.s.	5.30	5.73	4.00	5.98	6.23	6.50
Humidity of surface	**	3.78 ^ab^	3.88 ^ab^	4.23 ^a^	2.40 ^ab^	1.68 ^b^	3.30 ^ab^
Crumb residual	n.s.	1.98	2.48	1.65	1.73	1.18	0.88
Resistance to chewing	n.s	3.80	3.30	2.73	3.83	3.45	1.83
Juiciness	n.s.	2.90	4.78	3.40	2.05	1.20	2.85
Adhesiveness	n.s.	4.10	2.15	3.63	3.25	2.63	2.25
**Crust**								
Crispiness		*	5.25 ^ab^	4.20 ^ab^	7.35 ^a^	3.50 ^b^	4.35 ^ab^	7.03 ^a^
Hardness		n.s.	3.00	2.30	4.05	3.53	3.33	3.05
Smell intensity		n.s.	5.85	5.80	5.97	5.33	5.33	4.63
Salted taste		*	3.40 ^a^	3.48 ^a^	3.50 ^a^	2.03 ^b^	1.75 ^b^	2.38 ^b^
Toasted taste		**	3.78 ^b^	3.55^b^	6.95 ^a^	3.90 ^b^	4.63 ^ab^	5.70 ^ab^
Bitter taste		***	2.13 ^b^	2.25^b^	5.58 ^a^	1.98 ^b^	3.00 ^b^	4.80 ^a^
Aftertaste		n.s.	0.68	1.08	3.13	0.88	1.88	3.25

Notes: ^1^ Significance level *** *p* < 0.001, ** *p* < 0.01, * *p* < 0.05; ns: not significant (*p* > 0.05). Legend for symbol’s meaning: * = sample as main factor for ANOVA. Different letters indicate significant differences among samples. As expected, no significant differences were detected for the factor panellist.

**Table 9:** Water activity and colour parameters of bread slice samples at the appearance of mould (t = t_fin_).

	SB0	SB+P	SB+PA	YB0	YB+P	YB+PA
t _fin_ (days)	3	4	6	2	3	3
a_w_	0.949 ± 0.001	0.941 ± 0.001	0.940 ± 0.005	0.949 ± 0.001	0.950 ± 0.001	0.950 ± 0.005
L^*^	65.29 ± 1.61	54.60 ± 0.01	53.62 ± 1.01	68.75 ± 1.82	54.18 ± 0.94	55.54 ± 3.49
a^*^	0.68 ± 0.09	5.32 ± 0.01	5.03 ± 0.07	0.39 ± 0.06	3.28 ± 0.44	3.06 ± 0.08
b^*^	15.64 ± 0.46	9.38 ± 0.01	12.11 ± 0.16	14.71 ± 0.32	8.61 ± 0.24	11.85 ± 0.06
C^*^	15.66 ± 0.46	10.78 ± 0.10	13.11 ± 0.15	14.71 ± 0.33	11.11 ± 0.54	12.24 ± 0.15
H^*^	1.52 ± 0.20	1.16 ± 0.07	1.18 ± 0.02	1.54 ± 0.23	1.16 ± 0.07	1.32 ± 0.04

**Table 10:** Order zero kinetic parameters related to compressibility (mm) and weight decrease (%) of cooked bread during storage. Data are expressed as mean ± confidence interval.

	Compressibility (mm)			Weight Decrease (%)	
Sample	$k\pm \;c.i.\;(\mathrm{mm}\cdot {\mathrm{days}}^{-1})\;$	q (mm)	$\chi^{2}$	$k\pm \;c.i.\;({\mathrm{days}}^{-1})$	$\chi^{2}$
SB0	−0.43 ± 0.09	1.96 ± 0.09	0.46	−1.21 ± 0.01	5.5
SB+P	−0.49 ± 0.03	2.47 ± 0.12	6.71	−1.19 ± 0.01	3.66
SB+PA	−0.44 ±0.04	2.20 ± 0.16	4.60	−1.21 ± 0.01	0.84
YB0	−0.62 ± 0.12	2.31 ± 0.13	1.03	−1.40 ± 0.04	0.01
YB+P	−0.73 ± 0.14	3.68 ± 0.27	0.70	−1.30 ± 0.04	1.26
YB+PA	−0.63 ± 0.08	2.65 ± 0.16	0.12	−1.22 ± 0.01	0.94

## Supplementary Materials

The following are available online at https://www.mdpi.com/article/10.3390/foods10050942/s1, Table S1: UHPLC-HR-ESI-MS/MS data of potato constituents detected in sourdough bread + purple potatoes (SB+P) and baker’s yeast bread + purple potatoes (YB+P). Table S2: UHPLC-HR-ESI-MS/MS data of constituents from albedo detected in sourdough bread + purple potatoes + albedo (SB+PA) and baker’s yeast bread + purple potatoes + albedo (YB+PA). Table S3: UHPLC-HR-ESI-MS/MS data of potato anthocyanins (P) detected in sourdough bread + purple potatoes (SB+P), baker’s yeast bread + purple potatoes (YB+P), sourdough bread + purple potatoes + albedo (SB+PA) and baker’s yeast bread + purple potatoes + albedo (YB+PA). Table S4: Complete headspace compositions of cooked breads (w = whole or s = sliced) as a function of fortification and leavening agent used. Table S5: UHPLC-HR-ESI-MS/MS data of compounds a-f detected in the Citrus x aurantium albedo (A), but not found in bread samples enriched with A. Figure S1: UHPLC-HR-ESI-MS profiles of hydroalcoholic extracts of purple potatoes (P), shown in negative and positive ion mode, and Citrus x aurantium albedo (A). For peak data see Table S5.
