# Supplementary material for: Bread Fortified with Cooked Purple Potato Flour and Citrus Albedo: An Evaluation of Its Compositional and Sensorial Properties
Source: Foods. 2021 Apr 25;10(5):942. doi: 10.3390/foods10050942 (PMC8146928; doi:10.3390/foods10050942)

Figure S1. UHPLC-HR-ESI-MS profiles of hydroalcoholic extracts of purple potatoes (P), shown in negative and positive ion mode, and *Citrus x aurantium* albedo (A). For peak data see Table S5.

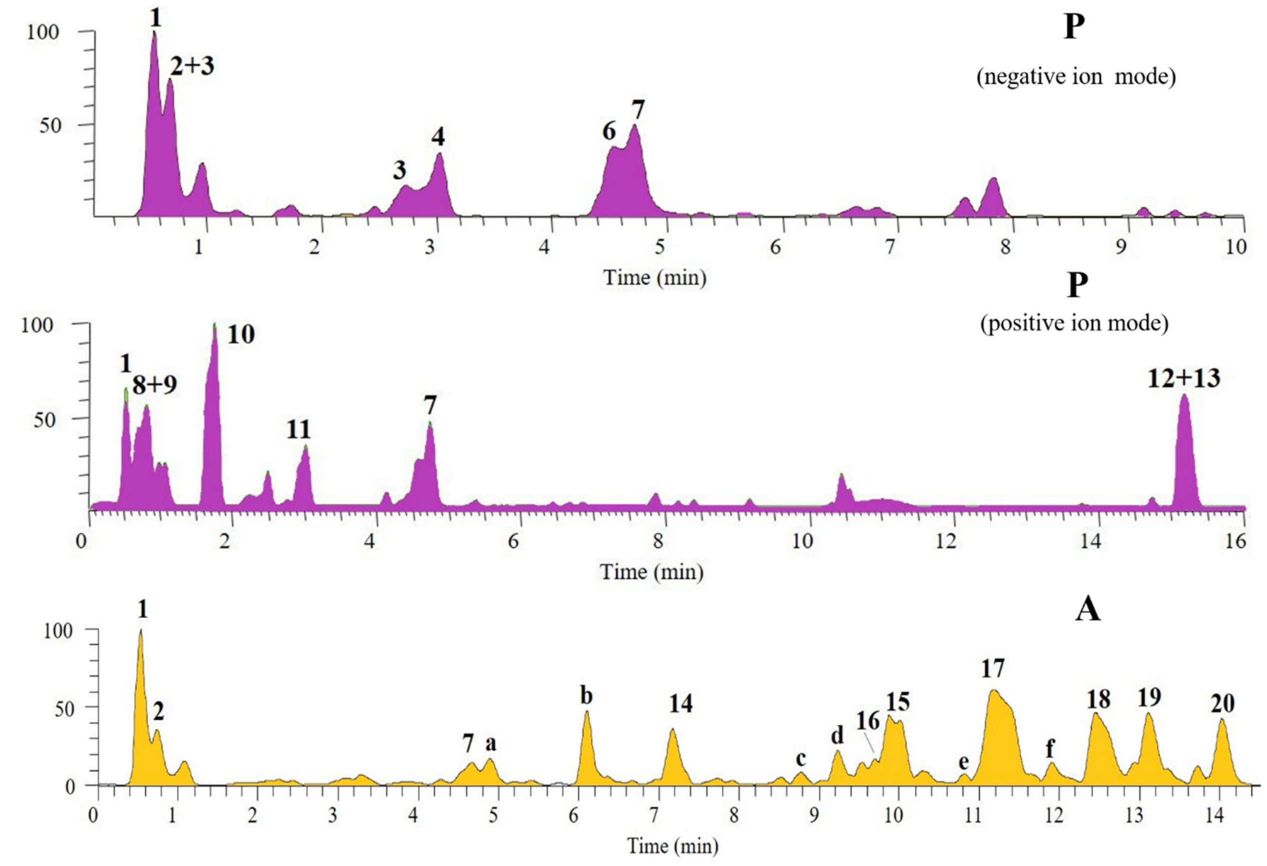

Supplement: Supplementary file 1 [file foods-10-00942-s001.zip › foods-1167723-supplementary/Figure S1.pdf]
