# Supplementary material for: Bread Fortified with Cooked Purple Potato Flour and Citrus Albedo: An Evaluation of Its Compositional and Sensorial Properties
Source: Foods. 2021 Apr 25;10(5):942. doi: 10.3390/foods10050942 (PMC8146928; doi:10.3390/foods10050942)
Supplement: Supplementary file 1 [file foods-10-00942-s001.zip › foods-1167723-supplementary/Table S1.pdf]

Table S1 UHPLC-HR-ESI-MS/MS data of potato constituents detected in sourdough bread + purple potatoes (SB+P) and baker's yeast bread + purple potatoes (YB+P).

**Peak***	**Compound**	t_R **(min)**	**HR-[M−H]⁻ (*m/z*)**	**HR-[M+H]⁺ (*m/z*)**	**Negative Product Ions (*m/z*)****	**Positive Product Ions (*m/z*)****	**Error (ppm)**
**1**	Saccharose	0.5	377.0857 ([M+HCOO]⁻)	381.0786 ([M+K]⁺)	341.10, 179.05	219.03	-1.84
**2**	Citric acid isomer I	0.6	191.0192		173.01, 129.02, **111.01**, 87.01, 85.03		-2.61
**3**	Citric acid isomer II	0.7	191.0192		173.01, 129.02, **111.01**, 87.01, 85.03		-2.61
**4**	Caffeoylquinic acid isomer I	2.9	353.0877		**191.05**, 179.03, 135.04		-0.28
**5**	Caffeoylquinic acid isomer II	3.0	353.0877		**191.05**, 179.03, 135.04		-2.23
**6**	Caffeic acid	4.5	179.0345		135.0444		
**7**	Caffeoylquinic acid isomer III	4.7	353.0877	355.1018	**191.05**, 179.03, 173.045, 135.04	**163.04**, 135.04, 89.04	-0.28
**8**	Leucine/isoleucine	0.6		132.1019		**86.09**, 69.07	0
**9**	Tyrosine	0.7		182.0811		**165.05**, 136.07, 123.04, 91.05	-3.29
**10**	Phenylalanine	1.7		166.0861		**120.08**, 103.05, 91.05	-0.60
**11**	Tryptophan	2.9		205.0970		**188.07**, 146.06, 118.07, 91.05	-0.49
**12**	α-Chaconine	15.3		852.5081		706.45, 544.37, 398.33	-2.58
**13**	α-Solanine	15.3		868.5036		722.44, 398.34	-1.96

*Peak numbers correspond with those in Figure 2. ** The base ion peaks generated in the ESI-MS/MS experiments are shown in bold.
