# Supplementary material for: Bread Fortified with Cooked Purple Potato Flour and Citrus Albedo: An Evaluation of Its Compositional and Sensorial Properties
Source: Foods. 2021 Apr 25;10(5):942. doi: 10.3390/foods10050942 (PMC8146928; doi:10.3390/foods10050942)
Supplement: Supplementary file 1 [file foods-10-00942-s001.zip › foods-1167723-supplementary/Table S2.pdf]

Table S2 UHPLC-HR-ESI-MS/MS data of constituents from albedo detected in sourdough bread + purple potatoes + albedo (SB+PA) and baker's yeast bread + purple potatoes + albedo (YB+PA).

**Peak***	**Compound**	t_R **(min)**	**HR-[M−H]$^-$ (*m/z*)**	**Negative Product Ions (*m/z*)****	**Error (ppm)**
	**Flavone *C*-glucosides**				
**14**	vicenin-2 (apigenin 6,8-di-*C*-glucoside)	7.2	593.1517	503.12, 473.11, 383.08, **353.07**	+1.01
	**Flavone *O*-glycosides**				
**16**	Apigenin 7-*O*-rutinoside/neohesperidoside	11.2	577.1567	413.01, **269.05**	+0.87
	**Flavanone *O*-glycosides**				
**15**	Eriocitrin (eriodictyol 7-*O*-rutinoside)	10.0	595.1671	459.11, 287.06, **151.00**, 135.04	+0.50
**17**	Naringin (naringenin 7-*O*-neohesperidoside)	11.5	579.1721	459.12, 271.06, **151.00**	+0.34
**18**	Neohesperidin (hesperetin 7-*O*-neohesperidoside)	12.6	609.1825	489.14, **301.07**, 286.05	+0.16
	**3-Hydroxy-3 methyl-glutaryl flavanone *O*-glycosides**				
**19**	Melitidin	13.2	723.2146	661.21, 621.18, 579.17, 459.12, 271.06, **151.00**	+0.69
**20**	Brutieridin	14.0	753.2249	691.22, 651.19, 609.18, **301.07**	+0.27

*Peak numbers correspond with those in Figure 3. ** The base ion peaks generated in the ESI-MS/MS experiments are shown in bold.
