# Supplementary material for: Bread Fortified with Cooked Purple Potato Flour and Citrus Albedo: An Evaluation of Its Compositional and Sensorial Properties
Source: Foods. 2021 Apr 25;10(5):942. doi: 10.3390/foods10050942 (PMC8146928; doi:10.3390/foods10050942)
Supplement: Supplementary file 1 [file foods-10-00942-s001.zip › foods-1167723-supplementary/Table S3.pdf]

Table S3 LC-HR-ESI-MS/MS data of potato anthocyanins (P) detected in sourdough bread + purple potatoes (SB+P), baker's yeast bread + purple potatoes (YB+P), sourdough bread + purple potatoes + albedo (SB+PA) and baker's yeast bread + purple potatoes + albedo (YB+PA).

**Peak***	**Compound**	t_R **(min)**	**HR-[M+H]+ (*m*/*z*)**	**Positive Product Ions (*m*/*z*)****	**Error (ppm)**
**21**	Malvidin 3-*O*-rutinoside-5-*O*-glucoside	2.0	801.2426	639.19, 493.13, **331.08**	-2.62
**22**	Malvidin 3-*O*-caffeoyl-rutinoside-5-*O*-glucoside	2.8	963.2739	801.22, 493.13, **331.08**	-2.60
**23**	Petunidin 3-*O*-caffeoyl-rutinoside-5-*O*-glucoside	2.9	949.2589	787.21, 479.11, **317.06**	-2.00
**24**	Petunidin 3-*O*-feruloyl-rutinoside-5-*O*-glucoside	3.1	963.2731	801.22, 479.12, **317.06**	-3.43
**25**	Petunidin 3-*O*-*p*-coumaroyl-rutinoside-5-*O*-glucoside	3.2	933.2643	771.21, 479.11, **317.06**	-1.71
**26**	Malvidin 3-*O*-*p*-coumaroyl-rutinoside-5-*O*-glucoside	3.4	947.2794	785.23, 493.13, **331.08**	-2.28
**27**	Malvidin 3-*O*-feruloyl-rutinoside-5-*O*-glucoside	3.5	977.2893	815.23, 493.13, **331.08**	-2.86

*Peak numbers correspond with those in Figure 4. **The base ion peaks generated in the ESI-MS/MS experiments are shown in bold.
