# Supplementary material for: Bread Fortified with Cooked Purple Potato Flour and Citrus Albedo: An Evaluation of Its Compositional and Sensorial Properties
Source: Foods. 2021 Apr 25;10(5):942. doi: 10.3390/foods10050942 (PMC8146928; doi:10.3390/foods10050942)
Supplement: Supplementary file 1 [file foods-10-00942-s001.zip › foods-1167723-supplementary/Table S5.pdf]

Table S5 UHPLC-HR-ESI-MS/MS data of compounds **a-f** detected in the *Citrus aurantium* albedo (A), but not found in bread samples enriched with A.

**Peak***	**Compound**	**t_R (min)**	**HR-[M-H]$^-$ (*m/z*)**	**Negative Product Ions (*m/z*)****	**Error (ppm)**
**a**	Caffeic acid glucoside	4.9	341.1239	**179.03**, 135.0	-0.29
**b**	Feruloyl acid glucoside	6.1	355.1035	193.05, **175.04**, 149.06	0.00
**c**	Lucenin-2 4′-methyl ether (diosmetin 6,8-di-*C*-glucoside)	8.9	623.1619	503.12, 413.09, 383.08, **312.06**	+0.16
**d**	Naringin glucoside	9.2	741.2254	579.17, **271.06**, 151.00	+0.94
**e**	Diosmetin 8-*C*-glucoside/6-*C*-glucoside	10.8	461.1091	361.08, 341.07, **298.05**	+0.43
**f**	3-Hydroxy-3-methyl-glutaryl-neoericitrin/eriocitrin	11.9	739.2099	595.17, 287.06, **151.00**	+1.08

*Peak numbers correspond with those in Figure 1S. **The base ion peaks generated in the ESI-MS/MS experiments are shown in bold.
